# Microbial and Metabolic Profiling of Obese and Lean Luchuan Pigs: Implications for Phenotypic Divergence

**DOI:** 10.3390/ani14142111

**Published:** 2024-07-19

**Authors:** Lihui Zhu, Shengwei Ma, Chuan He, Lan Bai, Weilong Tu, Xiao Wu

**Affiliations:** 1 Institute of Animal Husbandry and Veterinary Science, Shanghai Academy of Agricultural Sciences, Shanghai 201106, China; zhulihui@saas.sh.cn; 2 Key Laboratory of Agricultural Genetics and Breeding, Biotechnology Research Institute, Shanghai Academy of Agricultural Sciences, Shanghai 201106, China; mashengwei@webmail.hzau.edu.cn (S.M.); hechuan@saas.sh.cn (C.H.); bailan@saas.sh.cn (L.B.)

**Keywords:** phenotypic differentiation, gut microbiome, metabolomics, SCFAs

## Abstract

**Simple Summary:**

After approximately two decades of controlled breeding at the Shanghai Academy of Agricultural Sciences in China, Luchuan (LC) pigs have developed two distinct phenotypes: one with obesity (FLC) and the other with leanness (LLC). Since recent research suggests a link between microorganisms and the development of these different phenotypes, this study investigated the fecal microbiota profiles and serum metabolites of FLC and LLC pigs. We found significant changes in the microbiota and metabolites between the two groups. The correlation studies showed a significant link between the modified microbiota, metabolites, and the observed phenotypic differences in the LC pigs. Notably, *Jeotgalicoccus* was positively correlated with the body weight and chest circumference, but negatively correlated with certain metabolites like 2-mercaptobenzothiazole and N1-pyrazin-2-yl-4-chlorobenzamide, which were positively associated with *Bacteroides*. These findings provide strong evidence for a new connection between the gut microbiome, metabolome, and phenotypic variation in LC pigs.

**Abstract:**

Luchuan (LC) pigs are a Chinese breed renowned for their distinctive black and white coloring, superior meat quality and rapid reproduction, but their growth rate is slow. Over the course of approximately two decades of controlled breeding, the LC pigs maintained at the Shanghai Academy of Agricultural Sciences (Shanghai, China) have diverged into two phenotypes: one characterized by obesity (FLC) and the other by leanness (LLC). Recent studies indicate a correlation between microorganisms and the differentiation of host phenotypes. In this study, we examined the fecal microbiota profiles and serum metabolites of FLC and LLC pigs. The body weight, chest circumference, and alanine aminotransferase and aspartate aminotransferase enzyme activities were increased in the FLC pigs compared to the LLC pigs. Conversely, the levels of the *Fusobacterium* and *Streptococcus* genera were lower in the FLC pigs, while the number of *Firmicutes*, *Lactobacillus*, *Phascolartobacterium*, and *Rikenellaceae*_RC9_gut_group members were higher. A total of 52 metabolites were altered between the two groups, with many playing crucial roles in prolactin signaling, oocyte meiosis, and aldosterone-regulated sodium reabsorption pathways. The correlation analyses demonstrated a significant association between the modified microbiota and metabolites and the phenotypic variations observed in the LC pigs. Specifically, *Jeotgalicoccus* was positively correlated with the body weight and chest circumference, but was negatively correlated with metabolites such as 2-mercaptobenzothiazole and N1-pyrazin-2-yl-4-chlorobenzamide, which were positively associated with *Bacteroides*. These results provide compelling evidence for a novel relationship between the gut microbiome and metabolome in the phenotypic differentiation of LC pigs.

## 1. Introduction

Luchuan (LC) pigs are renowned for their high intramuscular fat content and backfat thickness compared with the Duroc breed, a prominent lean pig breed from the United States [1,2]. LC pigs display distinct characteristics such as small body size, stress resistance, reproductive performance, tolerance to rough feeding, and superior meat quality [1]. In China, the Shanghai Academy of Agricultural Sciences (Shanghai, China) has conserved a group of LC pigs imported from the Guangxi Zhuang Autonomous Region (Guangxi, China) in 2000. Over the course of the last two decades of selective breeding, the LC pigs have exhibited phenotypic differentiation resulting in two distinct morphological shapes. Specifically, a subset of LC pigs displaying a slender physique devoid of abdominal sagging were designated lean LC pigs (LLC), whereas another subset characterized by a larger body size, obesity, and abdominal contact with the ground were identified as fatty LC pigs (FLC). This raises the question of the underlying factors contributing to the observed phenotypic variation among the LC pigs subjected to identical feeding, environmental, and nutritional conditions.

The phenotype of an organism is determined by the interplay between its genotype and the environment, resulting in a set of physical and functional traits. The gut microbiota, a diverse microbial community inhabiting the host’s digestive tract, exerts a substantial influence on the host’s phenotype. The intricate ecosystem of the animal gut microbiota plays a crucial role in various physiological functions of the host, including food intake, metabolic regulation, immune system activation, and defense against infections [3]. Recent studies suggest that the microbiota play a key role in shaping host phenotypes, impacting various characteristics and functions. The interplay between metabolism and host phenotype is intricate, with the gut microbiota strongly influencing the host’s metabolic processes and overall health [4].

This relationship is bidirectional, with the host’s diet, lifestyle, and genetics shaping the composition and function of the microbiota, which in turn affects the host’s metabolic phenotype [4,5,6]. Host genetics are believed to play a significant role in shaping the composition of the gut microbiota [7]. Research indicates that various pig breeds exhibit distinct gut microbiota profiles, with Chinese domestic pigs displaying a greater diversity of intestinal flora than imported breeds including Duroc, Yorkshire, and Landrace [8,9]. Furthermore, variation in the gut microbiota has the potential to influence host phenotype, as interactions between hosts and their microbiota can impact genetic and phenotypic diversity. Specifically, the gut microbiota are thought to contribute to the overall genetic diversity by modulating gene expression [10]. This interaction can lead to variations in traits such as immune responses, metabolism, and even behavior, which are crucial for host adaptation and survival [10,11,12].

However, research on the role of individual variation in controlling bacterial communities and the influence of host phenotypic differentiation on microbial community formation is currently lacking. Furthermore, studies have demonstrated that the porcine gut microbiome reference catalogue contains more non-redundant genes shared between humans and pigs than between humans and mice, suggesting that pigs may serve as more suitable animal models than rats [10,13].

In order to examine the relationship between the microbiota and phenotypic variation in LC pigs, we assessed the differences in the intestinal microorganisms and serum metabolites in LLC and FLC pigs. Additionally, we explored the body habitus and blood physiology to identify correlations that may elucidate the connection between phenotypic variation and microbiota in LC pigs. This study provided foundational data for the development of models related to obesity-associated diseases.

## 2. Materials and Methods

### 2.1. Animal and Sample Collection

**Figure 1:**
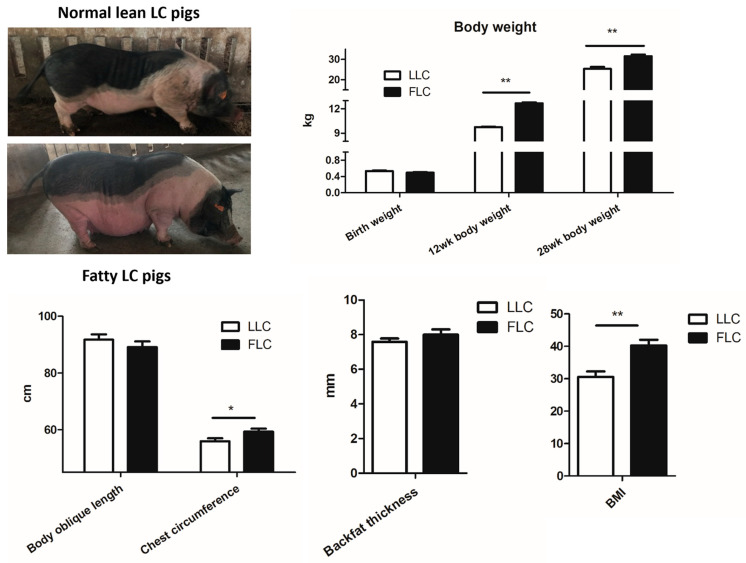
Phenotypic analysis of Luchuan pigs at 28 weeks of age. Data are presented as the mean ± SD values of each group (*n* = 12). Data were analyzed using Student’s *t*-test, and *p*-values were considered statistically significant at * *p* < 0.05 and ** *p* < 0.01. FLC, fatty Luchuan pigs; LLC, lean Luchuan pigs; BMI, body mass index. Pigs used in this study are offspring of the 50 female LC pigs.

At 25 weeks of age, 50 female LC pigs with specific characteristics (average weight: 30.31 kg, average body length: 91.07 cm, body mass index (BMI): 36.84) were selected from the Seed Protection Farm of Shanghai Academy of Agricultural Sciences (Shanghai, China) and divided into two groups: a lean body size group (LLC; BMI ≤ 36.84 and slender body shape) and an obese group (FLC; BMI > 36.84 and abdominal contact with the ground), determined by the degree of apparent obesity and body shape (Figure 1). Subsequently, these female LC pigs were used as breeding parents and were artificially inseminated with male LC pig parents for selective breeding. The female offspring pigs from the two groups were weighed at 0 days, 12 weeks, and 28 weeks of age. Additionally, at 28 weeks of age, 12 offspring were randomly selected from each group of FLC and LLC sows for measurement of body length, chest circumference, and collection of feces and blood samples for further analysis. Blood samples were collected from anterior vena cava and centrifuged at 3000 rpm for 15 min to obtain serum. For each pig, fresh feces within 12 h were collected in a piping bag and stored at −80 °C until further analysis. From birth to 28 weeks of age, these pigs were fed the same basal diet (Table S1) as that described by Pan et al. [14]. All pigs had free access to feed and water under the same housing conditions. BMI was calculated using the following equation:

BMI = Body weight (kg)/Body oblique length^2^ (m)

### 2.2. Inflammatory Cytokine Analysis

Levels of interleukin 2, 4, 8 (IL-2, IL-4, IL-8), immunoglobulin A, G, M (IgA, IgG, IgM), interferon γ (IFN-γ), and interferon α (IFN-α) in serum samples were quantified using a commercial ELISA kit (R&D, Minneapolis, MN, USA), while levels of albumin (ALB) and globulin (GLOB), as well as the activities of alanine aminotransferase (ALT), alkaline phosphatase (ALP), and glutamyl transpeptidase (GGT) in serum, were determined using a Hitachi 7100 automatic biochemical analyzer (Hitachi, Tokyo, Japan).

### 2.3. Microbiome Analysis

Fecal DNA was extracted using an E.Z.N.A. Stool DNA Kit (Omega Bio-tek, Norcross, GA, USA) following the manufacturer’s protocol. The V4 variable region of the 16S rRNA gene was PCR-amplified using universal primers 515F (5′-barcode- GTGCCAGCMGCCGCGG)-3′ and 907R (5′-CCGTCAATTCMTTTRAGTTT-3′). The amplified fragments were retrieved and sequenced using an Illumina HiSeqPE250 platform (conducted by Shanghai Biozeron Biological Co., Ltd., Shanghai, China). Initial sequences from sequencing were combined into a single sequence based on the overlap between PE reads. Quality control removed low-quality and spliced reads. Samples were distinguished by barcodes and primer sequences at the start and end of the sequence to obtain valid sequences, with the sequence orientation adjusted. Subsequently, operational taxonomic unit (OTU) clustering and species annotation were performed. Species annotation analysis was carried out using Mothur software (v1.31.2) [15], counting species with higher relative abundance and their proportions in each sample. Alpha and beta diversity were assessed using QIIME software (v1.9.1) [16]. Raw data were archived in the NCBI Sequence Read Archive database (PRJNA1095186).

### 2.4. Short-Chain Fatty Acid (SCFA) Measurement

Fecal SCFAs were measured as described previously [17], and detected as acid hydrazides at 400 nm. Briefly, samples were initially treated with 70% ethanol and centrifuged to isolate the supernatant. Following this, each sample was combined with 2-ethylbutyric acid as an internal standard and subsequently derivatised with pyridine. The reaction was then catalysed by 1-EDC-HCl and 2-NPH-HCl at a temperature of 60 °C, with the addition of potassium hydroxide and subsequent incubation at 60 °C for 20 min to halt the reaction. After cooling, the mixture was agitated and combined with an aqueous phosphoric acid solution and diethyl ether, followed by centrifugation to retrieve the supernatant. The resulting diethyl ether layer was then evaporated using nitrogen.

### 2.5. Untargeted Serum Metabolomics

The serum metabolome was analyzed using an untargeted approach at Shanghai Biozeron Biotechnology Co., Ltd. (Shanghai, China). The analysis was conducted using a Vanquish UHPLC system (Thermo Fisher Scientific, Germering, Germany) coupled with an Orbitrap Q ExactiveTM HF mass spectrometer (Thermo Fisher Scientific). Samples were homogenized with an 80% methanol aqueous solution to obtain water extractions, which were then analyzed on a Q Exactive HF-X liquid chromatography system (Thermo Fisher Scientific) with gradient elution on a Hypesil Gold column (100 × 2.1 mm, 1.9 μm; Thermo Fisher Scientific, Foster City, CA, USA) at a flow rate of 0.2 mL/min. Metabolic fragments were acquired in both positive and negative ion modes using a Vanquish UHPLC mass spectrometer (Thermo Fisher Scientific) operating in full scan mode from 100 to 1500 *m*/*z* with a capillary voltage set at 3.5 kV. Data were evaluated by unsupervised PCA, PLS-DA, and orthogonal partial least squares discriminant analysis (OPLS-DA). The variable importance in the projection (VIP) of the OPLS-DA model was calculated with a 200-permutation test and *p*-values were determined using Student’s *t*-test for single-dimensional statistical analysis. Metabolites with fold-change ≥ 1.5 || fold-change ≤ 1/1.5, VIP > 1, and *p* < 0.05 were considered distinct metabolites.

### 2.6. Statistical Analyses

The study group underwent testing for concentrations of SCFAs, with *p*-values between groups assessed using the Kruskal–Wallis rank sum test. A comparable analysis was conducted for inflammatory cytokines, with significance determined at *p* < 0.05. Spearman’s correlation coefficients were employed to examine the relationships between body weight, differential genera microbiota, and metabolites, with associations deemed significant if the absolute value of Spearman’s rank correlation coefficient (Spearman’s r) exceeded 0.6 and was statistically significant (*p* < 0.05). All statistical analyses were performed accordingly.

## 3. Results

### 3.1. Clinical Characteristics

As shown in Figure 1 and Table 1, the offspring pigs in the LLC and FLC groups exhibited distinct breed characteristics, with significant differences in the body weight, chest circumference, body mass index (BMI), and certain serum biochemical indicators (*p* < 0.05). The FLC pigs had a significantly higher body weight at 12 and 28 weeks, chest circumference, BMI, IgA, IgG, and IFN-γ levels than the LLC pigs (*p* < 0.05), while the IL-2 levels were lower in the FLC pigs (*p* < 0.05). Moreover, the activities of ALT andALP were higher in the FLC pigs (*p* < 0.05). There was no notable difference in the IL-4, IL-8, IgM, and IFN-α levels between the two groups, and, likewise, the ALB, GLOB, and GGT levels.

### 3.2. Microbiota Profiles Differ Significantly between LLC and FLC Pigs

**Figure 2:**
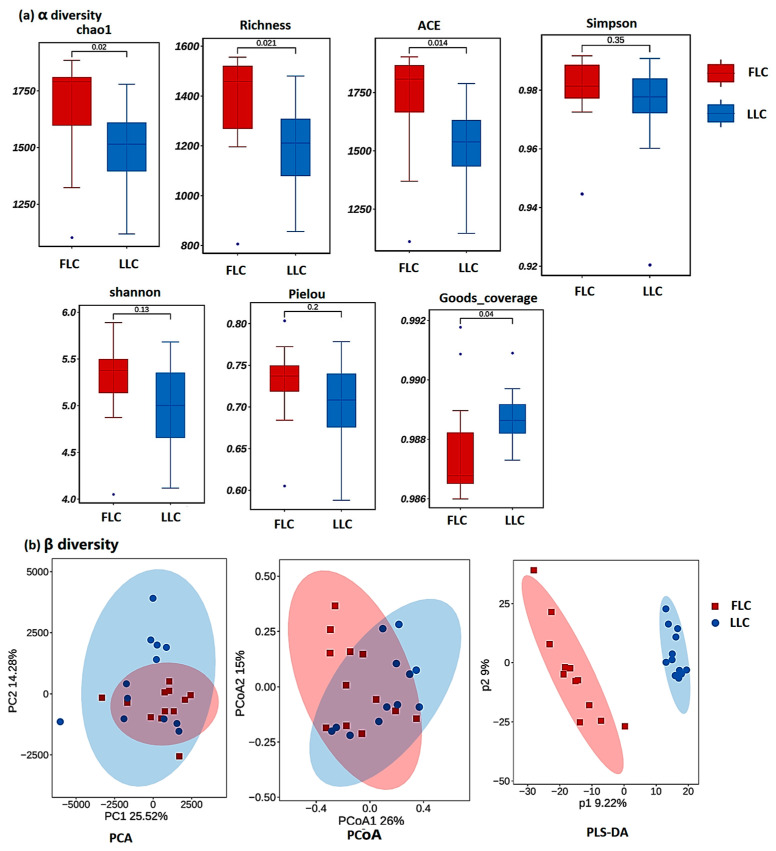
Alpha diversity and beta diversity analyses. (**a**) Alpha diversity indices of fecal bacterial communities in FLC and LLC pigs. (**b**) Beta diversity analysis of fecal bacterial communities in FLC and LLC pigs. Fecal samples of pigs were collected at 28 weeks of age. Twelve samples from each group were used for alpha diversity and beta diversity analyses. Twelve samples from each group were used for bacterial composition and diversity analyses. Data were analyzed using the Wilcoxon rank sum test. Feces were collected from the offspring of the LLC and FLC pigs. FLC, fatty Luchuan pigs; LLC, lean Luchuan pigs; PCA, principal component analysis; PcoA, principal coordinate analysis; PLS-DA, partial least squares discriminant analysis.

The alpha diversity of the fecal microbiota in the LLC and FLC pigs is illustrated in Figure 2a. An analysis of the alpha diversity using the Chao 1, Richness, and ACE indices gave consistent results. The Chao 1, Richness, and ACE indices of the fecal samples from the FLC pigs were significantly higher than those from the LLC pigs (*p* < 0.05), indicating a greater microbial diversity in the FLC pigs. The beta diversity analysis (Figure 2b) did not show a significant difference in the principal coordinate analysis (PCoA) and principal component analysis (PCA) between the two groups. The partial least squares discriminant analysis (PLS-DA) plot demonstrated a clear separation between the clusters of the FLC and LLC samples at both the species and genus levels. There was some overlap between the fecal samples from the LLC and FLC pigs, suggesting a degree of similarity in the bacterial communities within the same intestinal segment of the different pig breeds.

**Figure 3:**
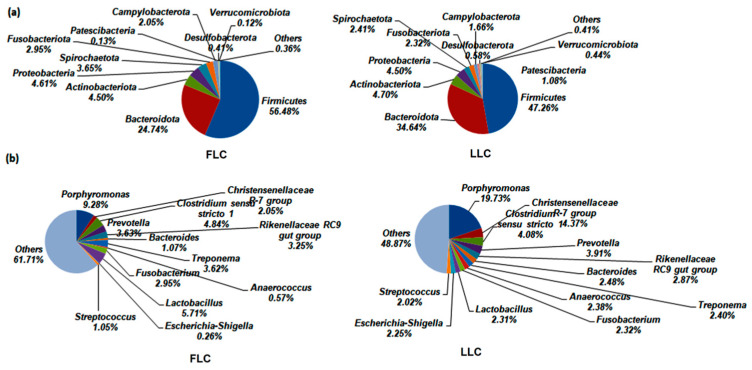
Fecal microbiota composition differs between FLC and LLC pigs. Relative abundance of bacteria at (**a**) phylum and (**b**) genus level. Fecal samples of the offspring of the LLC and FLC pigs were collected at 28 weeks of age. Twelve samples from each group were used for microbiota composition analysis. Values shown are the average of 12 samples from FLC and LLC pigs, respectively. FLC, fatty Luchuan pigs; LLC, lean Luchuan pigs.

**Figure 4:**
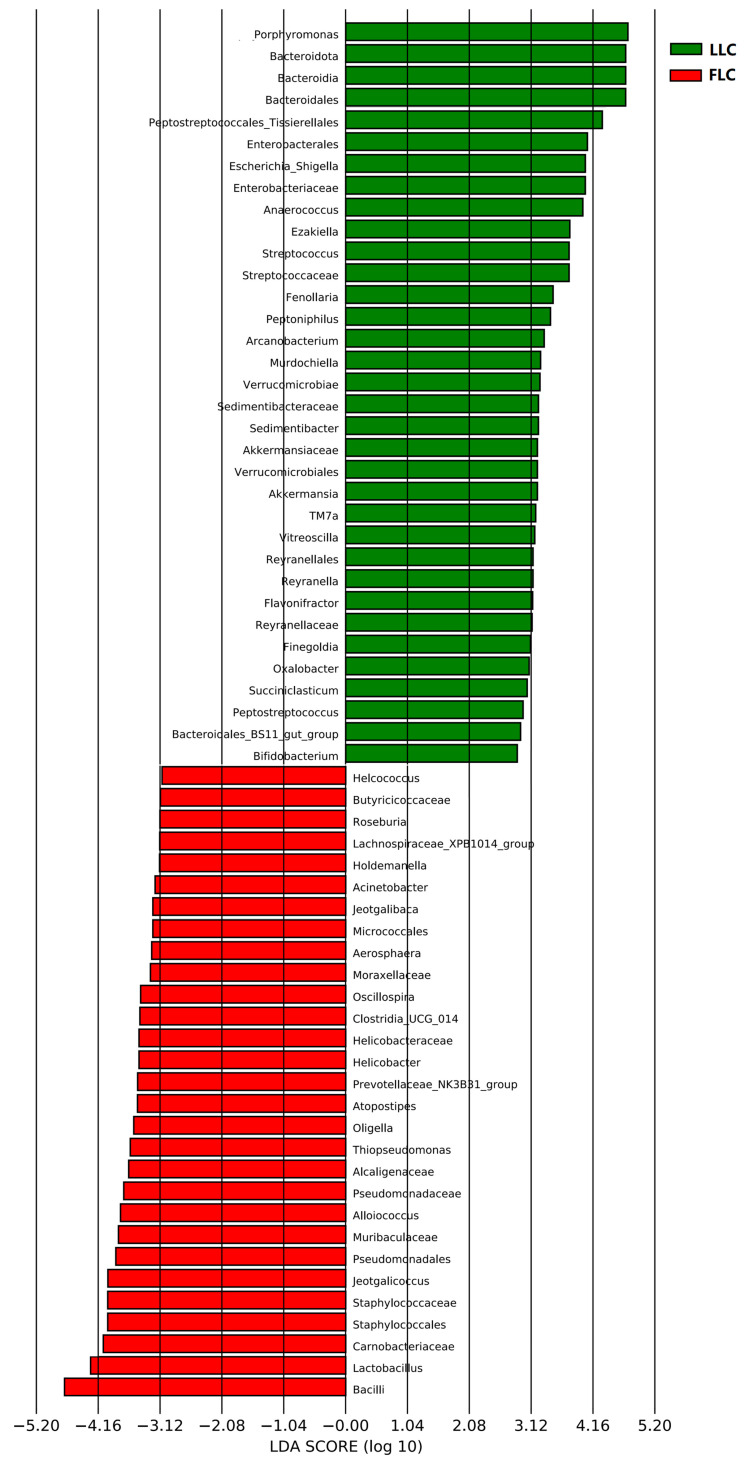
Differentially enriched taxa identified by linear discriminant analysis effect size (LEfSe) among the two groups. The length of the column represents the influence of significantly different species based on relative abundance (LDA scores [log10] > 3.1). Fecal samples of the offspring of the LLC and FLC pigs were collected at 28 weeks of age. Twelve samples from each group were used for microbiota composition analysis. FLC, fatty Luchuan pigs; LLC, lean Luchuan pigs.

The *Firmicutes* and *Bacteroidota* phyla in the fecal microbiota of the LLC and FLC pigs were found to be dominant, accounting for ~81% in both groups (Figure 3). The linear discriminant analysis effect size (LEfSe) was used to identify specific microbial communities that differed significantly between the two groups. It was observed that the LLC pigs had lower levels of *Firmicutes* and higher levels of *Fusobacteria* than the FLC pigs, with notable differences in abundance (47.26% vs. 56.48%, 2.95% vs. 2.32%, Figure 3a). Additionally, the relative abundance of *Bacteroidota* was higher in the LLC pigs than in the FLC pigs, although the difference was not statistically significant. At the genus level, *Lactobacillus* was more abundant in the FLC pigs, while *Porphyromonas* and *Streptococcus* were more prevalent in the LLC pigs. Furthermore, as shown in Figure 4, opportunistic pathogens such as *Escherichia*-*Shigella* and *Porphyromonas* were found to be overrepresented in the LLC group, while *Lactobacillus*, *Jeotgalicoccus*, *Prevotellaceae_NK3B31_group*, and *Bacilli* were predominant in the FLC group.

### 3.3. Metabolome Alterations between FLC and LLC Pigs

**Figure 5:**
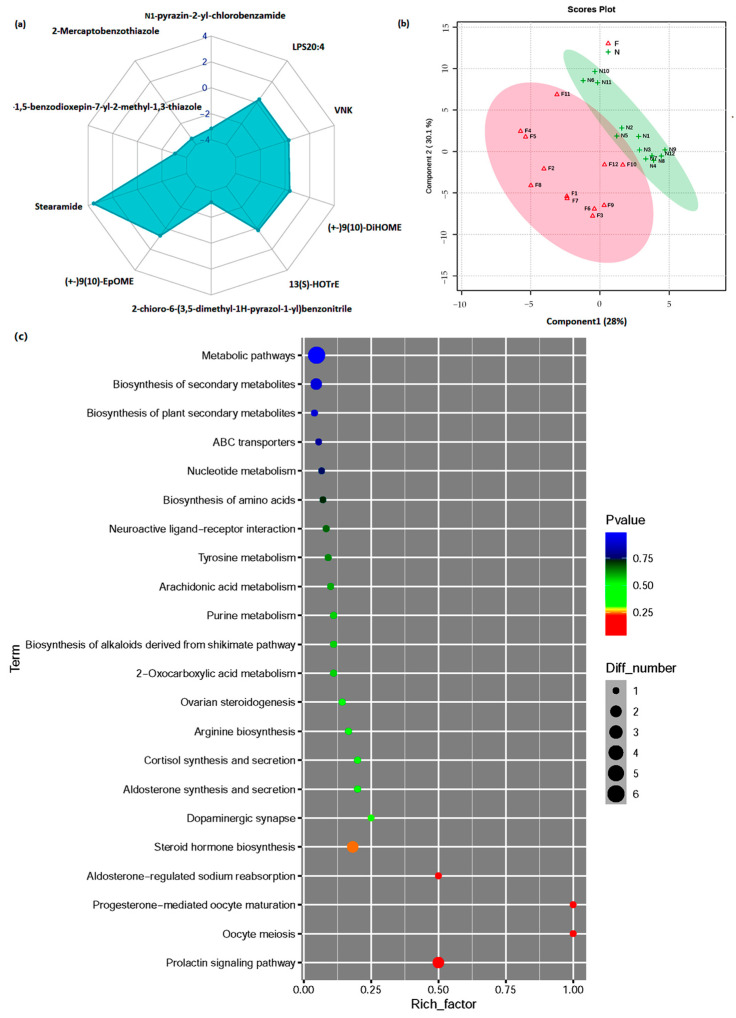
Differentially expressed metabolites in serum samples between FLC and LLC pigs. (**a**) PLS-DA score plots for FLC and LLC serum samples. (**b**) Radar plot of differentially expressed metabolites. (**c**) Pathway enrichment analysis of significantly elevated metabolites in serum of pigs according to Kyoto Encyclopedia of Genes and Genomes pathways of FLC and LLC models. Serum samples of offspring pigs were collected at 28 weeks of age. Twelve samples from each group were used for metabolite analysis. FLC, fatty Luchuan pigs; LLC, lean Luchuan pigs; PLS-DA, partial least squares discriminant analysis. F: offspring pigs from FLC group, N: offspring pigs from LLC group.

As illustrated in Table S2, the SCFA levels were generally increased in the feces of the FLC pigs. However, no significant differences were observed in the SCFAs between the two groups (*p* < 0.05). Butyric acid were more than four times higher in the feces of the FLC pigs than those of the LLC pigs. Furthermore, 52 named metabolites (26 detected in positive mode and 26 detected in negative mode), including 33 upregulated and 19 downregulated compounds, were found to be significantly altered between the FLC and LLC pigs (Table S3). Among these metabolites, OxPC (18:1-18:2 + 2O), stearamide, delta-17-6-keto prostaglandin F1alpha, and 2-amino-1,3,4-octadecanetriol showed increased levels, while 2-mercaptobenzothiazole, 2-chloro-6-(3,5-dimethyl-1*H*-pyrazol-1-yl) benzonitrile, and N1-pyrazin-2-yl-4-chlorobenzamide exhibited decreased levels in the serum of the FLC pigs compared to the LLC pigs (Figure 5a). PLS-DA was employed to confirm the distinct metabolic profiles between the two groups, with each sample showing a clear separation within its respective category (Figure 5b). A pathway enrichment analysis revealed that the primary prolactin signaling pathway, oocyte meiosis, and progesterone-mediated oocyte maturation were the top pathways affected in the FLC pigs compared to the LLC pigs (Figure 5c). These pathways are known to play a role in oocyte maturation.

### 3.4. Correlations between Growth Performance, Gut Microbiota, and Metabolites

**Figure 6:**
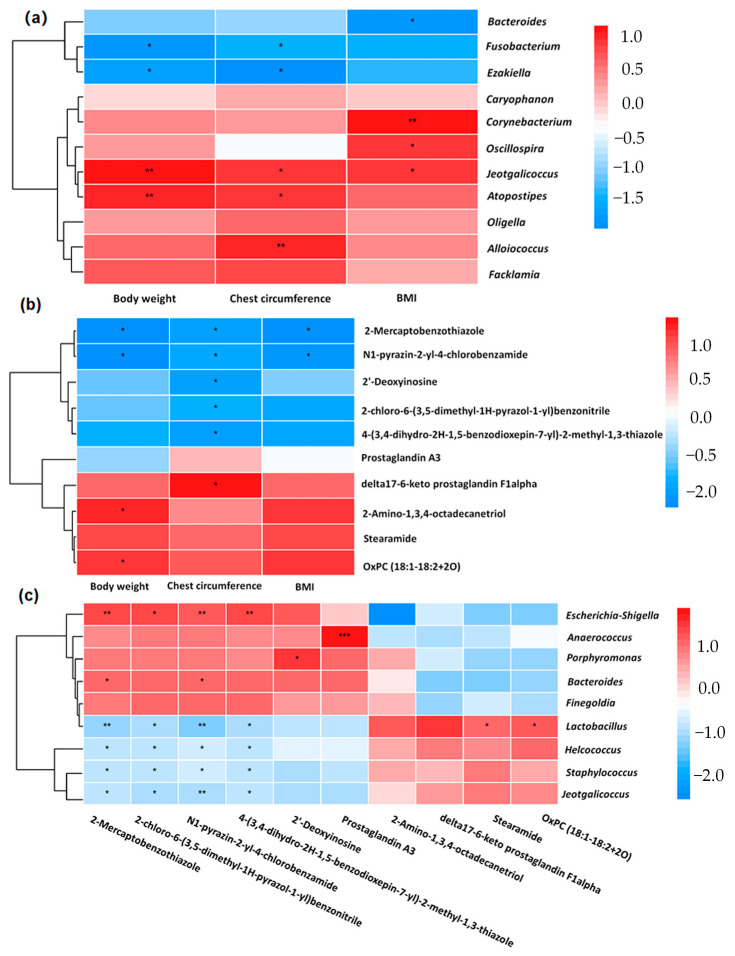
Correlations between (**a**) body weight and microbiota, (**b**) body weight and metabolites, and (**c**) microbiota and metabolites. Red represents positive correlations and blue represents negative correlations, *p*-values were considered statistically significant at * *p* < 0.05, ** *p* < 0.01, and *** *p* < 0.001. BMI, body mass index.

The correlation analysis revealed that the BMI was positively correlated with *Cornebacterium*, *Oscillospira*, and *Jeotgalicoccus*, but negatively correlated with *Bacteroides*. Additionally, the body weight and chest circumference showed positive associations with *Jeotgalicoccus* and *Atopostipes*, but negative associations with *Fusobacterium* and *Ezakiella* (*p* < 0.05; Figure 6a). Furthermore, the chest circumference was positively associated with delta17-6-keto prostaglandin F1alpha, but negatively associated with N1-pyrazin-2-yl-4-chlorobenzamide, 2’-deoxyinosine, 4-(3,4-dihydro-2*H*-1,5-benzodioxepin-7-yl)-2-methyl-1,3-thiazole, 2-mercaptobenzothiazole, and 2-chloro-6-(3,5-dimethyl-1*H*-pyrazol-1-yl) benzonitrile (*p* < 0.05). Moreover, the body weight and BMI were negatively associated with 2-mercaptobenzothiazole and N1-pyrazin-2-yl-4-chlorobenzamide (*p* < 0.05; Figure 6b). The analysis also identified four metabolites, N1-pyrazin-2-yl-4-chlorobenzamide, 2-chloro-6-(3,5-dimethyl-1*H*-pyrazol-1-yl) benzonitrile, 2-mercaptobenzothiazole and 4-(3,4-dihydro-2*H*-1,5-benzodioxepin-7-yl)-2-methyl-1,3-thiazole, that were downregulated in the FLC pigs, and were most negatively associated with the bacteria *Lactobacillus*, *Helcococcus*, *Staphylococcus*, and *Jeotgalicoccus*, but positively correlated with *Escherichia*-*Shigella* (*p* < 0.05). Additionally, 2-mercaptobenzothiazole and N1-pyrazin-2-yl-4-chlorobenzamide were found to be positively associated with *Bacteroides* (Figure 6c), and a positive relationship was observed between stearamide and OxPC (18:1-18:2 + 2O) and *Lactobacillus*.

## 4. Discussion

The gut microbiota are a complex community of microorganisms residing in the gastrointestinal tract that plays a significant role in shaping the phenotype of the host organism [18]. The phenotype encompasses physical, physiological, and behavioral traits. After over two decades of closed breeding, the FLC and LLC pigs in our study exhibited notable phenotypic differences. Specifically, the FLC pigs displayed a high level of fat accumulation resembling human obesity, with increased body weight, chest circumference, and BMI, as well as elevated ALT and ALP activities compared to the LLC pigs. To explore the potential contributions of the microbiota to phenotypic variations in the LC pigs, we conducted an analysis of the microbial diversity and metabolites in two distinct phenotypic groups. The results revealed significant changes in both the intestinal microbial community and metabolite profiles in the FLC pigs compared with the LLC pigs.

The gut microbiome structure in the LC pigs resembles that of various mammalian models, including humans and mice, with *Bacteroidetes*, *Firmicutes*, and *Proteobacteria* as the dominant microbial communities [3,10,19,20], similar to the core pig microbiota suggested by Holman et al. [21]. Interestingly, a high presence of *Lactobacillus* was observed in the FLC pigs, contrary to findings in obese pig models such as Ossabaw pigs, where the *Lactobacillus* abundance was decreased [22]. Studies on obese mice and humans show a higher proportion of *Firmicutes* and *Bacteroidetes*, with a positive correlation between increased *Firmicutes*, energy intake, fat storage, and *Bacteroidetes* reduction and fat loss [23,24,25]. However, conflicting results exist in some studies on humans and pigs regarding the proportion of *Firmicutes* and *Bacteroidetes* in obese and lean individuals [26]. Recent systematic reviews on bariatric surgery patients indicate that obese individuals have distinct gut microbiota profiles compared to non-obese individuals, with a higher prevalence of phyla including *Firmicutes* and *Proteobacteria*, orders such as *Clostridiales*, and genera like *Lactobacillus* in obese individuals [27]. Conversely, the relative abundance of *Bacteroidetes*, *Faecalibacterium*, and *Bifidobacterium* tends to be lower in obese individuals [28], consistent with our findings in the FLC pigs. Additionally, an increase in *Treponema* spp. has been noted in relation to adiposity in different pig breeds such as Jinhua, Mangalica, and Göttingen mini pigs [29,30,31]. Herein, an increase in the genus *Treponema* was also observed in the FLC pigs. These findings suggest that there is specificity in the bacterial species that are related to obesity, where bacteria within the same genus may have contrasting roles in obesity. This complexity in obesity metabolism mechanisms is further supported by Cheng et al. (2022) [32].

Nowadays, it is widely accepted that the gut microbiota plays a significant role in influencing the host’s metabolism through their impact on energy extraction from food, fat storage, and the regulation of hormones related to appetite and energy expenditure. Changes in the composition of the gut microbiota have been linked to metabolic disorders like obesity [33]. In the current study, the FLC pigs displayed an obese phenotype, and their gut microbiota exhibited a somewhat unique response to obesity. Specifically, we observed that certain genera, such as *Jeotgalicoccus*, were positively correlated with the BMI, body weight, and chest circumference in the LC pigs, suggesting a potential association with the phenotypic differences in these pigs, but this needs further investigation. Importantly, evidence has shown that the relationship between the gut microbiota and the host is bidirectional, with the host’s environment and genetics influencing the microbiota composition, and conversely, the microbiota impacting the host’s phenotype in various ways [4]. The genetic make-up of the host can impact the gut microbiota composition, potentially playing a role in obesity [34]. The variability observed in our results highlighted the unique characteristics of the microbial population in LC pigs, influenced by the genetics of the host or external factors, as evidenced in another pig model [35].

Researchers suggest that the gut microbiota may produce numerous metabolites, which are absorbed by the intestinal epithelium and enter the bloodstream, ultimately affecting the body’s metabolic health [32]. Metabolites are crucial in shaping phenotypes, and understanding their composition is essential for unravelling the mechanisms underpinning specific biological properties. In the present study, 52 differential metabolites were successfully identified between the sera of the FLC and LLC pigs, comprising organic acids and derivatives, fatty acyls, benzenoids, and lipids. OxPC (18:1-18:2 + 2O) was found to be the most upregulated in the FLC pigs, followed by stearamide, which is also upregulated in the plasma of humans with nonalcoholic fatty liver disease [36]. Additionally, the gut microbiota convert indigestible dietary fibre into SCFAs through fermentation. SCFAs, including acetic, propionic, and butyric acids, play crucial roles in regulating nutrient absorption, hormone production, and energy metabolism in the intestine [20]. The diversity of microbial communities in feces is closely linked to the range of metabolites present [37]. Obese individuals show elevated SCFA levels, primarily attributed to increased *Firmicutes* abundance [33]. We found that changes in the fecal metabolites were caused by various bacteria, and the richness of the gut microbial species also affected serum metabolites and led to changes in body metabolism. For instance, a positive correlation was observed between the chest circumference and delta-17-6-keto prostaglandin F1alpha, while a negative association was noted with four downregulated metabolites including 2-mercaptobenzothiazole and N1-pyrazin-2-yl-4-chlorobenzamide in the serum of the FLC pigs. This suggested that the metabolic capabilities changed during the phenotypic differentiation of the LC pigs, potentially linked to their phenotypic changes. Moreover, the serum levels of stearamide and delta-17-6-keto prostaglandin F1alpha positively correlated with the abundance of the beneficial microbes *Lactobacillus* and *Roseburia*, implying a positive role of these upregulated metabolites in the phenotypic differentiation of the LC pigs. Metabolism and host phenotype are intricately connected, with the host’s metabolic processes significantly influenced by the gut microbiota. Notably, a negative relationship was found between the chest circumference and four downregulated metabolites in the FLC pigs, underscoring the potential impact of these downregulated metabolites on the phenotypic differentiation of LC pigs.

While we identified associations between the body weight, differential microbiota, and metabolites, understanding how certain core bacteria in LC pigs contribute to shaping phenotypic differentiation under the same nutrition and management regime is still in its infancy. Additionally, the exact functions of most metabolites remain largely unknown, limiting our ability to extract more useful information from this study. Further research is needed to explore the associations between altered metabolites and phenotypic changes in LC pigs. Given the significant role of the gut microbiota in regulating obesity, it is crucial to investigate the connection between obesity and the gut microbiota. Future research should focus on elucidating the mechanisms by which the gut microbiota contribute to obesity, and assessing the safety and effectiveness of potential treatments that restore the gut microbiota balance. Additionally, there is a need to explore the potential of utilizing LC pigs as disease models.

## 5. Conclusions

Our study revealed significant alterations in the microbiota and serum metabolites of two groups of LC pigs. We observed a decrease in the abundance of *Fusobacterium* and *Streptococcus* at the genus level in the FLC pigs, along with an increase in *Firmicutes*, *Lactobacillus*, *Phascolartobacterium*, and *Rikenellaceae*_*RC9*_gut_group compared to the LLC pigs. Furthermore, we identified 52 named metabolites involved in key pathways such as prolactin signaling, oocyte meiosis, progesterone-mediated oocyte maturation, and aldosterone-regulated sodium reabsorption, showing differential expression between the serum samples from the two groups. These changes in microbiota and metabolites were closely linked to the phenotypic differences observed in the LC pigs. Specifically, certain microbiota such as *Jeotgalicoccus* were positively correlated with increased body weight, BMI, and chest circumference, but were negatively associated with specific metabolites such as N1-pyrazin-2-yl-4-chlorobenzamide and 2-chloro-6-(3,5-dimethyl-1H-pyrazol-1-yl) benzonitrile. In conclusion, our results suggested that variations in gut microbial composition and metabolites play a significant role in the phenotypic diversity of LC pigs.

## Figures and Tables

**Table 1:** Analysis of serum biochemical indicators in FLC and LLC pigs.

Item	LLC	FLC	*p* Value
IL-2 (ng/L)	36.55 ± 0.95 ^c^	32.69 ± 0.91 ^a^	0.01
IL-4 (ng/L)	42.7 ± 1.11	41.85 ± 1.03	0.58
IL-8 (ng/L)	47.94 ± 1.19	44.63 ± 1.14	0.06
IgA (mg/L)	0.68 ± 0.00 ^a^	0.89 ± 0.01 ^c^	0.00
IgG (mg/L)	126.55 ± 2.48 ^a^	185.35 ± 2.86 ^c^	0.00
IgM (mg/L)	6.77 ± 0.28	7.12 ± 0.32	0.42
IFN-γ (ng/L)	65.25 ± 1.48 ^a^	70.31 ± 1.77 ^b^	0.04
IFN-α (ng/L)	255.13 ± 4.43	261.25 ± 4.50	0.34
ALT (U·L^−1^)	52.34 ± 1.16 ^a^	69.23 ± 1.89 ^c^	0.00
ALP (U·L^−1^)	173.42 ± 3.34 ^a^	255.89 ± 4.67 ^c^	0.00
GGT (U·L^−1^)	36.95 ± 1.18	39.45 ± 0.94	0.11
ALB (g·L^−1^)	41.46 ± 1.15	43.95 ± 1.17	0.14
GLOB (g·L^−1^)	28.97 ± 0.44	28.98 ± 0.89	0.11

Different letters indicate significant differences compared to the control (Student’s *t* test; adjacent letters indicate *p* < 0.05 and spaced letters indicate *p* < 0.01). Results are shown as mean ± SD values. IL-2, interleukin 2; IL-4, interleukin 4; IL-8, interleukin 8; IgA, immunoglobulin A; IgG, immunoglobulin G; IgM, immunoglobulin M; IFN-γ, interferon γ; IFN-α, interferon α; ALB, albumin; GLOB, globulin; ALT, alanine aminotransferase; GGT, glutamyl transpeptidase; ALP, alkaline phosphatase; FLC, fatty Luchuan pigs; LLC, lean Luchuan pigs.

## Data Availability

Raw data on microbiomes and metabolomes are deposited in NCBI under the SRA database with accession No. PRJNA1095186 and Metabo-Lights with accession No. MTBLS10235.

## Supplementary Materials

The following supporting information can be downloaded at https://www.mdpi.com/article/10.3390/ani14142111/s1; Table S1: The nutritional composition of the diet used in this study (air-dried basis); Table S2: Short-chain fatty acids in the feces of FLC and LLC pigs; Table S3: Differentially expressed metabolites in serum samples between FLC and LLC pigs.
