# Supplementary material for: Microbial and Metabolic Profiling of Obese and Lean Luchuan Pigs: Implications for Phenotypic Divergence
Source: Animals (Basel). 2024 Jul 19;14(14):2111. doi: 10.3390/ani14142111 (PMC11273426; doi:10.3390/ani14142111)
Supplement: Supplementary file 1 [file animals-14-02111-s001.zip › animals-3089166-supplementary.pdf]

Supplementary: Microbial and Metabolic Profiling of Obese and Lean Luchuan Pigs: Implications for Phenotypic Divergence

Lihui Zhu [1], Shengwei Ma [2], Chuan He [2], Lan Bai [2], Weilong Tu [1,*] and Xiao Wu [2,*]

[1] Institute of Animal Husbandry and Veterinary Science, Shanghai Academy of Agricultural Sciences, Shanghai 201106, China
[2] Key Laboratory of Agricultural Genetics and Breeding, Biotechnology Research Institute, Shanghai Academy of Agricultural Sciences, Shanghai 201106, China
* Correspondence: tuweilong@saas.sh.cn (W.T.); wuxiao@saas.sh.cn (X.W.)

Tabel S1 The nutritional composition of the diet used in this study (air dry basis)

Ingredient	Contents (%)
Corn, 8%	57.00
Fermented soybean meal, 47%	11.00
Expanded soybean	10.00
Wheat bran	18.00
Soybean oil	0.40
NaCl	0.25
Monocalcium phosphate	1.00
Limestone	0.70
L-Lys HCl	0.41
DL-Met	0.10
L-Thr	0.14
Premix[1]	1.00
Total	100
Nutrition level[2]	
Metabolic energy (Kcal/Kg)	11.98
Crude protein (%)	14.92
Crude fiber (%)	2.82
Crude ash (%)	11.11
Calcium (%)	0.83
Total phosphorus (%)	0.66
Lysine (%)	0.71

[1]Provided vitamin and mineral premix per kg of diet: vitamin A = 2400 IU; vitamin D3 = 2800 IU; vitamin E = 200 IU; vitamin K3 = 5 mg; vitamin B12 = 40 μg; vitamin B1 = 3 mg; vitamin B2 = 10 mg; niacin = 40 mg; pantothenic acid = 15 mg; folic acid = 1 mg; vitamin B6 = 8 mg; biotin = 0.08 mg; Fe ($FeSO_4 \cdot H_2O$) = 120 mg; Cu ($CuSO_4 \cdot 5H_2O$) = 16 mg; Mn ($MnSO_4 \cdot H_2O$) = 70 mg; Zn ($ZnSO_4 \cdot H_2O$) = 120 mg; I (CaI_2O_6) = 0.7 mg; Se (Na_2SeO_3) = 0.48 mg.

[2]All indicators are measured values except for Metabolic energy

Table S2 Short-chain fatty acids in the fecal of FLC and LLC pigs

Item	LLC (mg/g)	FLC (mg/g)	Fold change (FLC/LLC)	*P*-value
Isobutyric acid	0.101±0.026	0.267±0.167	2.649	0.336
Acetic acid	6.159±1.349	13.633±7.846	2.214	0.358
Butyric acid	0.112±0.069	0.503±0.445	4.479	0.394
Isovaleric acid	0.100±0.021	0.250±0.127	2.471	0.257
Valeric acid	0.049±0.020	0.145±0.103	2.983	0.372
Caproic acid	0.010±0.00	0.117±0.001	1.367	0.166
Propionic acid	0.731±0.229	1.938±1.627	2.646	0.470
Total	7.262±1.568	16.744±10.292	2.646	0.372

FLC, fatty Luchuan pigs; LLC, lean Luchuan pigs. Results are shown as mean ± SD values.

Table S3 Differentially expressed metabolites in serum samples between FLC and LLC pigs

Name	Formula	Molecular Weight	RT [min]	log2(F/L)	*P*-value	VIP
OxPC (18:1-18:2+2O)	C44 H82 N O10 P	861.58	11.13	4.14	0.01	1.81
Stearamide	C18 H37 N O	283.29	10.34	3.60	0.00	3.29
delta17-6-keto prostaglandin F1alpha	C20 H32 O6	414.23	6.47	2.23	0.01	1.59
2-Amino-1,3,4-octadecanetriol	C18 H39 N O3	317.29	6.08	2.11	0.05	1.90
N-METHYL (-)EPHEDRINE	C11 H17 N O	179.13	7.10	1.41	0.02	2.18
(+-)9(10)-EpOME	C18 H32 O3	296.23	7.66	1.12	0.00	3.12
(+/-)9-HpODE	C18 H32 O4	312.23	7.23	1.05	0.02	2.00
NQH	C15 H23 N7 O6	397.17	4.99	0.94	0.01	2.30
Dibutyl sebacate	C18 H34 O4	314.25	7.36	0.92	0.00	2.68
tetranor-PGFM	C16 H26 O7	312.15	5.73	0.91	0.00	1.97
octadec-9-ynoic acid	C18 H32 O2	262.23	8.37	0.83	0.00	2.36
(+-)9(10)-DiHOME	C18 H34 O4	314.25	7.36	0.83	0.00	2.67
(1E,4E)-1,5-bis(4-methoxyphenyl) penta-1,4-dien-3-one	C19 H18 O3	316.10	7.49	0.81	0.02	2.24
FAHFA (18:2/20:4)	C38 H62 O4	582.46	10.02	0.81	0.00	2.59
VNK	C15 H29 N5 O5	359.21	7.37	0.76	0.00	2.70
16-Hydroxyhexadecanoic acid	C16 H32 O3	272.23	7.97	0.76	0.01	2.42
LPS 20:4	C26 H44 N O9 P	545.27	8.54	0.76	0.00	2.33
Progesterone	C21 H30 O2	314.23	7.95	0.75	0.00	2.48
C-6 NBD ceramide	C30 H49 N5 O6	557.36	5.00	0.75	0.02	1.97
(+/-)12(13)-DiHOME	C18 H34 O4	296.23	7.95	0.75	0.00	2.60
5-[5-(ethylsulfonyl)-2-hydroxyanilino]-5-oxopentanoic acid	C13 H17 N O6 S	332.10	7.20	0.74	0.04	2.04
LPS 18:1	C24 H46 N O9 P	523.29	11.03	0.74	0.01	1.89
2-Aminoadipic acid	C6 H11 N O4	161.07	1.40	0.73	0.03	1.87
2-(1H-imidazol-1-ylmethyl)-3,4-dihydronaphthalen-1(2H)-one oxime	C14 H15 N3 O	241.12	5.14	0.73	0.03	1.73

12-oxo Phytodienoic Acid	C18 H28 O3	292.20	7.58	0.71	0.01	2.10
12-epi Leukotriene B4	C20 H32 O4	336.23	7.15	0.69	0.05	1.32
5-OxoETE	C20 H30 O3	318.22	8.08	0.68	0.02	2.28
13(S)-HOTrE	C18 H30 O3	294.22	7.62	0.66	0.00	2.82
12,13-EODE	C18 H32 O3	296.23	8.09	0.64	0.02	2.23
HNH	C16 H22 N8 O5	406.16	5.17	0.64	0.03	1.97
(+-)13-HODE	C18 H32 O3	296.23	9.25	0.64	0.02	2.07
FAHFA (18:1/20:3)	C38 H66 O4	586.49	10.58	0.63	0.01	2.25
Levodopa	C9 H11 N O4	197.07	1.40	0.61	0.01	2.31
Dihydrothymine	C5 H8 N2 O2	128.06	1.38	(0.64)	0.00	2.28
Glycerol-3-phosphate	C3 H9 O6 P	172.01	1.41	(0.69)	0.03	1.66
2-Methoxyresorcinol	C7 H8 O3	140.05	5.33	(0.72)	0.02	1.68
5'-Deoxy-5'-(Methylthio)Adenosine	C11 H15 N5 O3 S	297.09	5.14	(0.78)	0.00	2.45
N-Acetylornithine	C7 H14 N2 O3	174.10	1.40	(0.81)	0.02	1.94
2-(4-fluorophenyl)-5-(4-methyl-1,2,3-thiadiazol-5-yl)-1,3,4-oxadiazole	C11 H14 N4 O S	250.09	1.40	(0.82)	0.00	2.16
L-Homocitrulline	C7 H15 N3 O3	189.11	1.40	(0.85)	0.01	2.16
4-[2-(2-oxo-1-imidazolidinyl)ethyl]-1lambda~6~,4-thiazinane-1,1-dione	C9 H17 N3 O3 S	248.10	6.14	(0.86)	0.01	1.64
Inosine	C10 H12 N4 O5	268.08	3.55	(0.88)	0.02	1.72
Cortisone	C21 H28 O5	360.19	5.97	(0.91)	0.01	1.73
2-Oxindole	C8 H7 N O	133.05	5.66	(0.94)	0.04	1.25
11-trans Leukotriene C4	C30 H47 N3 O9 S	1250.61	5.04	(1.01)	0.00	2.62
Pizotifen	C19 H21 N S	295.15	7.19	(1.21)	0.05	1.44
Prostaglandin A3	C20 H28 O4	314.19	7.69	(1.34)	0.01	2.06
2'-Deoxyinosine	C10 H12 N4 O4	252.09	4.95	(1.50)	0.00	2.28
4-(3,4-dihydro-2H-1,5-benzodioxepin-7-yl)-2-methyl-1,3-thiazole	C13 H13 N O2 S	247.06	6.10	(2.18)	0.00	3.17
2-chloro-6-(3,5-dimethyl-1H-pyrazol-1-yl)benzonitrile	C12 H10 Cl N3	231.05	5.03	(2.26)	0.00	2.94

N1-pyrazin-2-yl-4-chlorobenzamide	C11 H8 Cl N3 O	255.02	6.24	(2.26)	0.00	3.24
2-Mercaptobenzothiazole	C7 H5 N S2	166.99	6.23	(2.51)	0.00	3.28
